# Discovery of novel coumarin derivatives as potent and orally bioavailable BRD4 inhibitors based on scaffold hopping

**DOI:** 10.1080/14756366.2019.1587417

**Published:** 2019-03-18

**Authors:** Zhimin Zhang, Lili Gu, Beibei Wang, Wenhai Huang, Yanmin Zhang, Zhen Ma, Shenxin Zeng, Zhengrong Shen

**Affiliations:** a Key Laboratory of Neuropsychiatric Drug Research of Zhejiang Province, Institute of Materia Medica, Zhejiang Academy of Medical Sciences, Hangzhou, PRChina;; b School of Basic Science, China Pharmaceutical University, Nanjing, PR China

**Keywords:** Rational drug design, BRD4 bromodomain, synthesis, biological evaluation

## Abstract

The bromodomain and extra-terminal (BET) bromodomains, particularly BRD4, have been identified as promising therapeutic targets in the treatment of many human disorders such as cancer, inflammation, obesity, and cardiovascular disease. Recently, the discovery of novel BRD4 inhibitors has garnered substantial interest. Starting from scaffold hopping of the reported compound dihydroquinazolinone (PFI-1), a series of coumarin derivatives were designed and synthesised as a new chemotype of BRD4 inhibitors. Interestingly, the representative compounds **13** exhibited potent BRD4 binding affinity and cell proliferation inhibitory activity, and especially displayed a favourable PK profile with high oral bioavailability (*F* = 49.38%) and metabolic stability (T_1/2_ = 4.2 h), meaningfully making it as a promising lead compound for further drug development.

## Graphical Abstract

**Figure:**
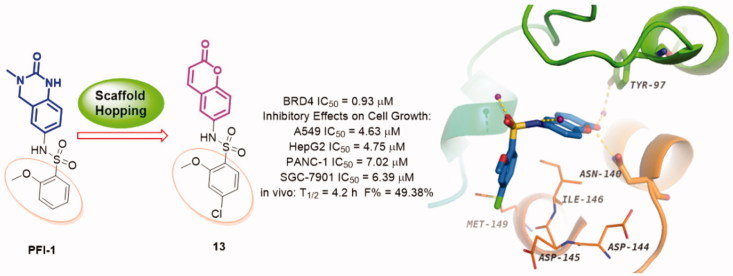

## Introduction

**Figure 1.**
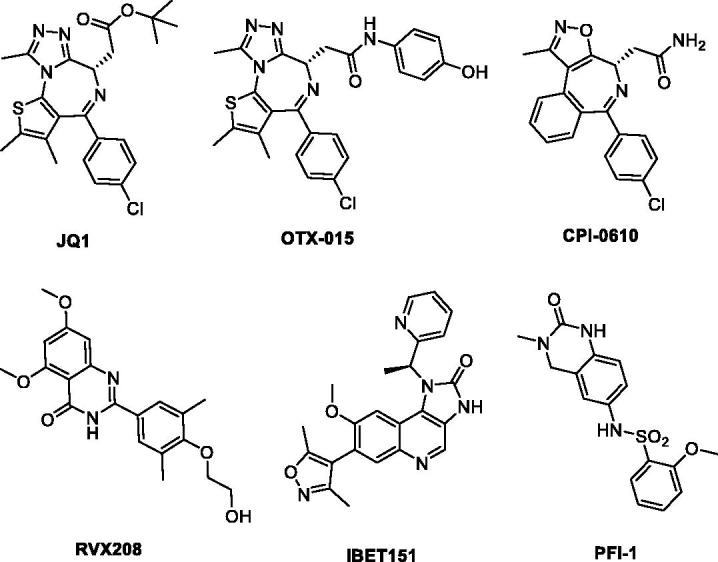
Representative, previously reported bromodomain inhibitors.

The bromodomain and extra-terminal (BET) family proteins, mainly including BRD2, BRD3, BRD4, and BRDT, are a kind of epigenetic regulatory proteins that selectively bind to acetylated lysines (KAc) of histone, thus translate chromatin status into transcription activation through RNA polymerase II and play critical roles in the regulation of gene transcription^1–3^. Among the BET family proteins, BRD4 has not just received a lot of attention, but been the most extensively studied one. The KAc residues bind into a hydrophobic pocket of the BRD4 proteins specifically with conserved asparagine (Asn140), also forming a water molecule-mediated hydrogen bonding interactions with tyrosine (Tyr97)^4^ ^,^ ^5^. Small molecule BRD4 inhibitors can block the binding, which leads to profound disruption of transcriptional programmes resulting in therapeutic potential in several conditions, such as inflammation and oncologic diseases^6–10^. In the last few years, a number of high-affinity small molecule ligands of BRD have been identified (Figure 1)^11–14^, wherein JQ-1 was the first reported potent BRD4 inhibitor and has been employed widely to evaluate its therapeutic potential in a great many preclinical human disease models^15^. Several JQ-1 analogues, such as OTX-015^16–18^ and CPI-0610^19^ ^,^ ^20^ have subsequently advanced into clinical trials for the treatment of solid tumours, haematological malignancies and other forms of human cancer. It was also worthy of attention that PFI-1, a dihydroquinazolinone compound reported to be a potent BRD inhibitor obtained through optimization of a fragment-derived hit^21^. However, the solubility and pharmacokinetics of PFI-1 are suboptimal^22^. Notwithstanding the above discovery, potent BRD4 inhibitors with novel chemotypes are still in high demand for only limited BRD4 inhibitors available in clinical trials.

**Figure 2.**
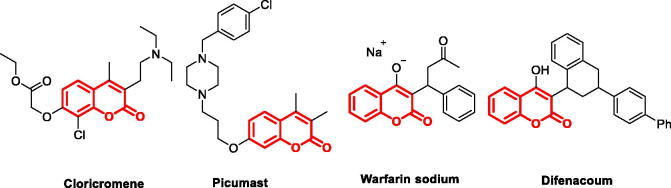
Structures of approved coumarin-containing drugs.

The natural products, normally with unique chemical structure, have long played an important role in drug discovery^23^. Coumarin (benzo-α-pyrone) skeleton found in nature has been considered as a privileged pharmacophore ascribed to the ability to exert noncovalent interactions (hydrogen bonds, hydrophobic, van der Waals force, metal coordination, and electrostatic interactions, etc.), with the various active sites in therapy targets^24^ ^,^ ^25^. Coumarins skeleton have gained momentous attention in the last decades as a lead structure for the discovery of orally bioavailable anti-cancer, anti-HCV, anti-HIV, anti-Alzheimer, and anti-inflammatory agents^26–29^. Several coumarin-based derivatives, such as Cloricromene^30^, Picumast^31^, Warfarin sodium^32^, and Difenacoum^33^ (Figure 2, coumarin skeleton marked in red) were approved for therapeutic purposes in clinic.

Scaffold hopping can not only provide an alternative strategy for BRD4 inhibitor design, but also potentially lead the discovery of potent BRD4 inhibitors with novel and diverse chemotypes. The advantages of scaffold hopping, combining with the pharmacophore property of coumarin and the skeleton modifiability of PFI-1 much interest us to explore structurally novel BRD4 inhibitors. Herein, we reported the rational design, syntheses, and structure-activity relationship (SAR) exploration of novel coumarin heterocycle derivatives as potent BRD4 inhibitors *via* the scaffold hopping of PFI-1. Furthermore, *in vitro* anti-tumour test and *in vivo* preliminary pharmacokinetics study of representative compound also were described in this article.

## Results and discussion 

### Design of new BRD4 inhibitors containing a coumarin scaffold

**Figure 3.**
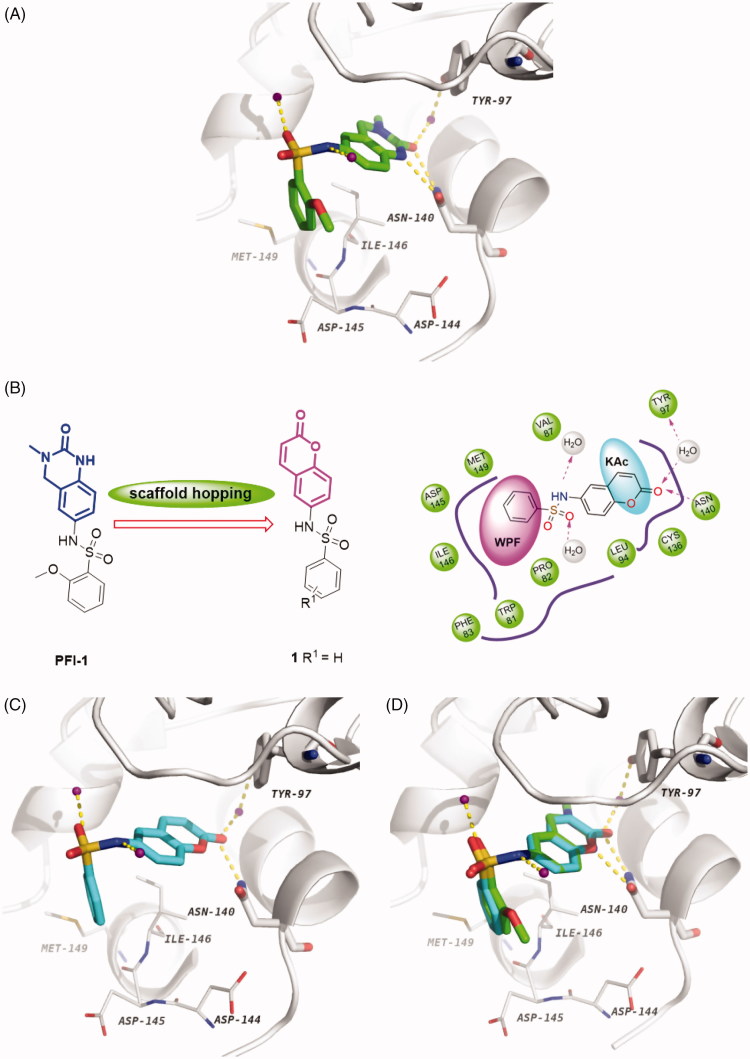
(A) Crystal structure of BRD4 BD1 bound to PFI-1 (PDB ID: 4E96). The protein is shown as a light gray cartoon and PFI-1 is shown as sticks (carbon atoms in green, oxygens in red, nitrogens in blue and sulfurs in brown). (B) Design concept of new BRD4 inhibitors. (C) The docking model of **1** with BRD4 BD1 (carbon atoms in cyan). (D) Superimposition of PFI-1 (green carbon atoms) and **1** (cyan carbon atoms) in their putative bioactive conformations.

Scaffold hopping is a powerful and promising strategy for drug discovery^34^. It has been extensively used for the design of structurally novel bioactive molecules^35–38^, which generally incorporates ring opening, ring closure, heterocycle replacement, and shape/topology-based scaffold hopping^39^ ^,^ ^40^. Herein, we applied the heterocycle replacement and shape-based scaffold hopping strategy towards PFI-1, a BRD inhibitor developed by Pfizer Worldwide R&D. First, we analysed the binding mode of PFI-1 binds to the BRD4 bromodomain. X-ray crystallographic analysis reveals that PFI-1 binds to BRD4 with three key hydrogen bonds interactions, which are displayed in Figure 3(A). The carbonyl oxygen and NH group of the dihydroquinazolinone forms two critical hydrogen bonds with the conserved residue Asn140. Moreover, the carbonyl oxygen interacts with the conserved residue Tyr97 *via* a water-mediated hydrogen bond. The anisole group occupies the WPF (formed by residues W97, P98 and F99) shelf and forms hydrophobic interaction with Asp145, Ile146, and Met149. The sulphonamide forms two additional hydrogen bonds with two water molecules. With this structural information in mind, we hypothesised that rational replacement of the dihydroquinazolinone core *via* coumarin skeleton based on scaffold hopping would be tolerated (Figure 3(B)). Thus, a series of novel coumarin derivatives were designed. As shown in Figure 3(C), the docking mode of coumarin derivative **1** binds to BRD4 was consistent with that of PFI-1. The alignment results depicted in Figure 3(D) also indicated that **1** almost took the same interaction conformation as PFI-1 except that the hydrogen-bond interaction between NH and Asn140 was lost.

### SAR studies of coumarin derivatives

With the understanding of binding conformation, our next work is to explore the SAR of the coumarin derivatives. We focused on the hydrophobic WPF shelf to develop compounds with improved affinity for BRD4. In order to optimise the interactions towards WPF shelf, diverse substituents at the R position were designed to investigate the chemical space for improving the activity. To ensure the R group extends to the WPF shelf and forms hydrophobic interactions with residues located there, we maintained the sulphamide linker. Thus, compounds **1 − 16** with R groups of aromatic groups, alkyl or cycloalkyl were designed and synthesised (Table 1). We preferentially evaluated the phenyl group. Compound **1** characterised by phenyl group has shown moderate BRD4 binding activity with an IC_50_ value of 6.59 μM in the AlphaScreen assay. When alkyl or cycloalkyl groups were used to occupy the WPF shelf, *a* > 3-fold decrease in affinity was found (Table 1, compounds **2**–**5**), probably due to a reduction of hydrophobic interactions with WPF shelf. Generally, the cycloalkyl group compounds (compounds **2** and **3**) were more tolerated than the chain alkyl compounds (**4** and **5**). Next, we paid our attention back to aromatic groups. An extensive SAR exploration was then conducted: displaying that diverse substitution at different positions of the phenyl group in **1** may lead to various effects on the BRD4 affinity. Our study showed that compounds bearing chlorine, methoxy group or tertiary butyl group at the *para*-position were tolerable (compounds **6**–**8**). The inhibitory activities of **6**–**8** were similar to **1** with IC_50_ values of 2.97, 7.31, and 5.09 μM, respectively. The *para*-chlorine substituted analogue **6** was somewhat more potent than **7** and **8**, which indicated that electron-withdrawing groups at the *para*-position might be favourable. We further investigated the impact of cyano group on *meta*-position, compound **9** maintained the similar potency at an IC_50_ value of 5.03 μM, which suggested that substitution at *meta*-position was also feasible. Surprisingly, the enhanced potency was achieved when substitutions were introduced at the *ortho*-position (compounds **10** and **11**). The *ortho-methoxy group* analogue **11** showed significant increase with an IC_50_ value of 0.98 μM and is approximately 7-fold more potent than **1**. Compound **11** was more potent than **10**, which may due to the advisable sulphamide conformation caused by the larger methoxyl group on *ortho*-position. With the results that mono-substituent at *para*-position or *ortho*-position was favourable; we attempted to merge these two positions as bi-substituent. The resulting compound **12** and **13** displayed potent activity with IC_50_ values of 1.77 and 0.93 μM, respectively. It is interesting that compound **12** was slightly more potent than **10**, while **13** was slightly more potency than **11,** which may ascribe to greater lipophilicity because of the chlorine atom. For example, the lipophilic efficiency (LipE, −lg (IC_50_) − logP) of **13** and **11** was 3.2 and 3.9. For more understanding the SAR, we further replaced the phenyl ring of **1** by thienyl group, the resulting derivative **14** showed low binding activity. The replacement by larger aromatic group also led to a reduction of affinity (compounds **15** and **16**).

**Table 1.** Structure-activity relationship of the R substitutions on synthesized coumarin derivatives. 
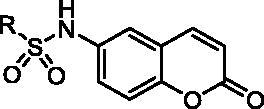

Comp. No.	R	BRD4 IC_50_ (μM)^a^	cLogP^b^	LE^c^
1		6.59	2.43	0.35
2		19.02	2.83	0.31
3		17.9	2.73	0.33
4		38.53	1.86	0.34
5		30.04	2.39	0.33
6		2.97	2.55	0.34
7		7.31	3.18	0.33
8		5.09	4.26	0.30
9		5.03	2.19	0.32
10		2.21	2.88	0.36
11		0.98	2.07	0.37
12		1.77	3.60	0.35
13		0.93	2.82	0.36
14		9.26	2.16	0.35
15		12.23	3.60	0.28
16		18.42	2.68	0.28
JQ1		0.06		
PFI-1^d^		0.22		

^a^The IC_50_ values were tested by the AlphaScreen assay; ^b^cLogP values were calculated using ChemBiodraw Ultra14.0; ^c^LE (Ligand Efficiency) = 1.4 (pIC_50_/heavy atoms); ^d^The IC_50_ value was reported by Fish et al.^22^.

**Figure 4.**
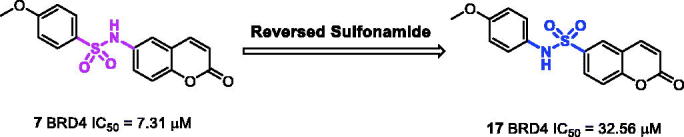
The design and synthesis of reversed sulfonamide compound **17**.

The x-ray crystal structure of PFI-1 bind to BRD4 revealed that the sulphonamide linker provides a specific angle turn for *N*-substitution to reach into WPF shelf. We were curious about that whether a reversed sulphonamide could achieve the same effect. We designed and synthesised a reversed sulphonamide compound **17** based on compound **7** (Figure 4). Unfortunately, **17** showed almost 5-fold decrease in potency compared with **7**.

### Docking study of the preferred configuration of representative compound 13

**Figure 5.**
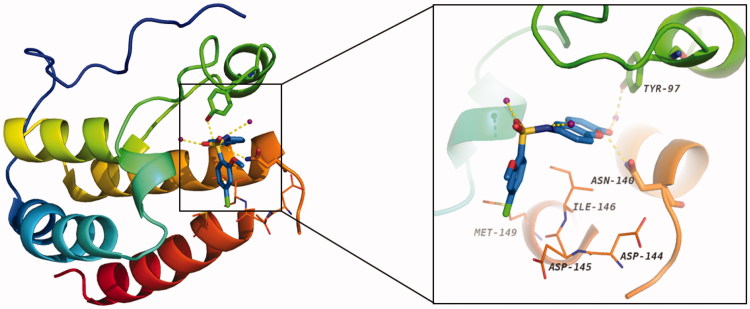
The docking model (PDB ID: 4E96) of **13** with BRD4 BD1 (carbon atoms in blue). Water molecule is shown as purple sphere, and the hydrogen bonds are denoted by gold dash lines.

To disclose the structural basis for the high binding affinity of compound **13** to the BRD4 protein, we conducted the docking study of **13** in a complex with BRD4 (Figure 5). As expected, the coumarin moiety of **13** forms two hydrogen bonds with Asn140 and Tyr97 *via* a conserved water molecule in the KAc binding site of BRD4. The 4-chloro-2-methoxybenzene group occupies the hydrophobic WPF shelf and forms hydrophobic interactions with Met149, Asp144, Asp145, and Ile146. The sulphonamide forms two additional hydrogen bonds with two water molecules.

#### Evaluation of the inhibitory effects on cell growth

**Figure 6.**
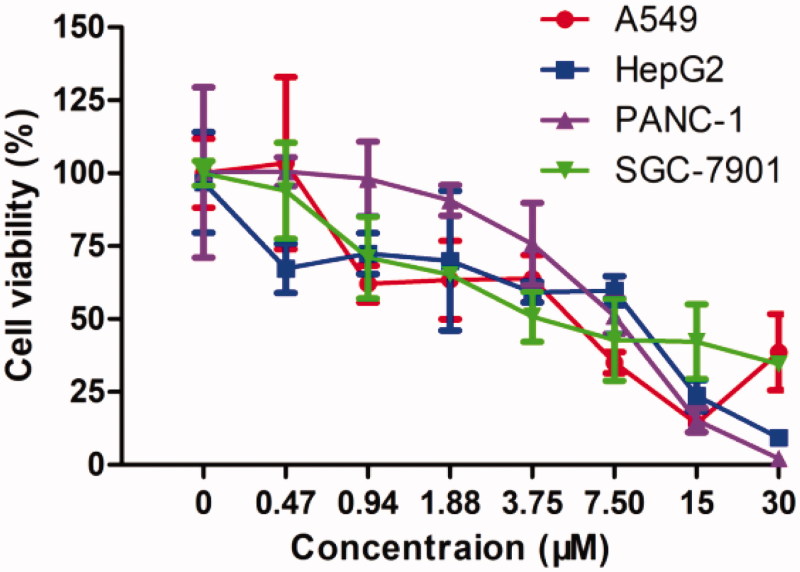
Dose–response curves of **13** in incubation with cancer cell lines (mean ± SD, *n* = 3).

The representative compound **13** was next evaluated for its effects on the survival of human lung adenocarcinoma A549 cells, hepatocellular carcinoma HepG2 cells, pancreatic carcinoma PANC-1 cells, and gastric adenocarcinoma SGC-7901 cells with an MTT assay. The data obtained were summarised in Table 2 and dose–response curves were provided in Figure 6. Results showed that **13** potently inhibits the proliferation in these four cell lines, with IC_50_ values of 4.63, 4.75, 7.02, and 6.39 μM, respectively. Overall consideration of the data from the above assays, **13** has good profiles for further evaluation.

**Table 2.** Anti-proliferation effects of **13** against four cell lines.

Cancer type	Cell line	IC_50_ (μM)
Lung adenocarcinoma	A549	4.63
Hepatocellular carcinoma	HepG2	4.75
Pancreatic carcinoma	PANC-1	7.02
Gastric adenocarcinoma	SGC-7901	6.39

### Effect of 13 on cells morphology

**Figure 7.**
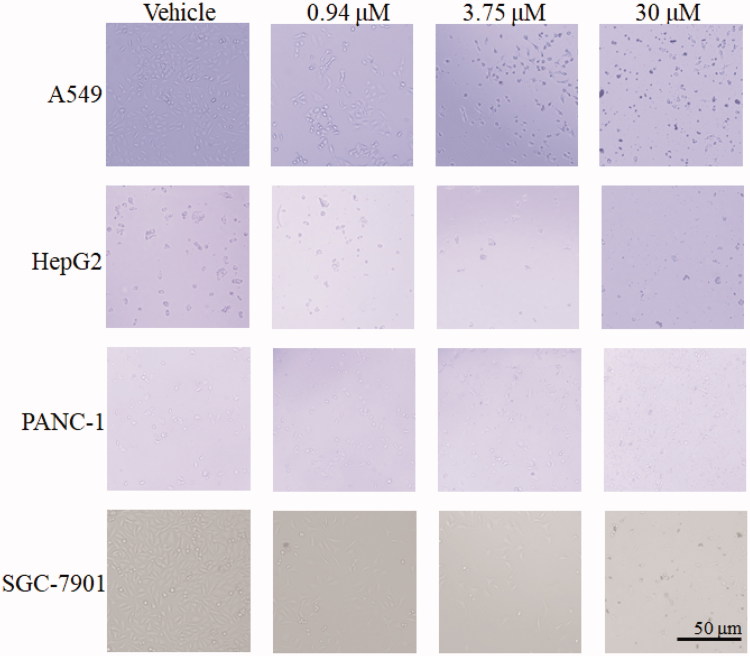
Effect of **13** on A549, HepG2, PANC-1 and SGC-7901 cells morphology. Cells after treating with **13** under relevant concentrations for 24 h were observed by invert/phase contrast microscopy.

As shown in Figure 7, no abnormality was observed in the SGC-7901 cells, PANC-1 cells, HepG2, and A549 cells from the control, with oval or short fusiform, adhered to the wall and liked paving stone shape. Cells in **13**-treated groups, with increased of dose, change became more obviously, contour was gradually clear, cell diopter strengthened, cells gradually became smaller and round, shrinking into the spherical, part of the cells was broken, and then fell off or suspended.

### Assessment of pharmacokinetic (PK) properties for 13

**Figure 8.**
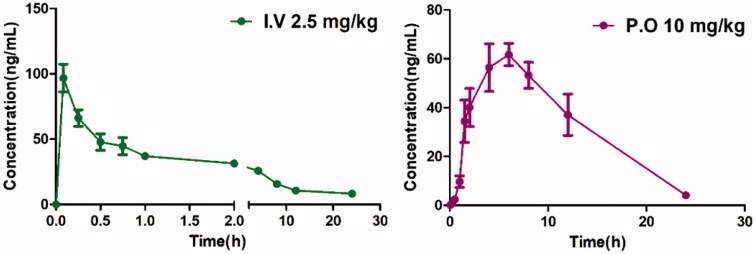
The plasma concentration-time curve of **13**.

To assess the potential of this series of compound *in vivo*, we evaluated **13** as a representative for its preliminary pharmacokinetics in Sprague-Dawley (SD) rats; the resulted data were listed in Table 3. The plasma was collected after a single oral dose of 10 mg/kg or an i.v. dose of 2.5 mg/kg (Figure 8). Compound **13** demonstrated preferable PK properties with a *T*_1/2_ value of 4.2 h and a *T*_max_ of 5.87 h. In addition, in the oral administration mode, **13** displayed good drug exposure with an AUC value of 790.65 μg/L*h and resulted in excellent oral bioavailability (49.38%), which is higher than that of PFI-1 (32%)^22^.

**Table 3.** Intravenous (i.v.) and oral (p.o) pharmacokinetic profiles of compounds 1**3** in rats.

Parameters	i.v. (2.5 mg/kg)	SD	p.o (10 mg/kg)	SD
AUC_(0–t)_ (μg/L*h)	400.87	32.41	790.65	55.47
AUC_(0–∞)_ (μg/L*h)	607.74	226.07	816.46	64.99
*T*_1/2_ (h)	–	–	4.2	0.29
*T*_max_ (h)	0.083	0	5.87	0.95
MRT_0–t_ (h)	7.9	0.81	8.5	0.66
CL_z_ (L/h/kg)	4.46	1.4	12.30	10.93
V_d_ (L/kg)	84.28	28.10	74.28	5.19
C_max_(μg/L)	96.87	10.56	61.7	4.6
F (%)	–	–	49.38	5.45

*The statistical significance is meaningless due to the different dosages between PO and IV.

### Chemistry

**Scheme 1.**
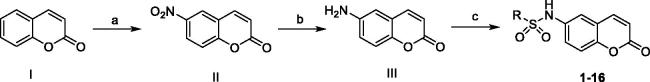
The synthetic route of compounds. Reagents and conditions: (a) HNO_3_/H_2_SO_4_; (b) SnCl_2_/HCl; (c) pyridine, sulphuryl chloride, rt.

The methodology employed for the synthesis of the coumarin derivatives was demonstrated in Scheme 1. The commercially available 2*H*-chromen-2-one (I) reacted with concentrated nitric acid in sulphuric acid under 0–5 °C to produce compound **II**, which was then reduced in ammonium chloride and iron powder system to give the amino intermediate **III**. The target compounds were (**1–16**) were obtained by sulphonylation reactions between **III** and various sulphonyl chlorides in pyridine, which act as solvent, as well as the deacid reagent.

## Conclusion

In this study, we have designed and synthesized a series of coumarin-containing compounds based on scaffold hopping with the goal of improving the oral bioavailability of PFI-1 and obtaining entirely new chemotype of BRD4 inhibitors. Our efforts have led to the discovery of a representative compound **13** (4-chloro-2-methoxy-*N*-(2-oxo-2*H*-chromen-6-yl)benzenesulfonamide), which binds to BRD4 with nanomolar affinities and shows low micromolar potencies in cell growth inhibition against four human cancer cell lines. Significantly, it achieves excellent oral bioavailability (*F* = 49.38%) and is metabolically stable (*T*_1/2_ = 4.2 h), which is preferable than PFI-1 (*F* = 32%). The obtained data suggested that **13** possesses a promising BRD4 inhibitory and orally bioavailable characteristic, worth of making it as a lead compound for further drug development.

## Experimental section

### BRD4 binding assay

The binding affinity was evaluated by a using AlphaScreen technology FRET assay. The biotinylated peptide binding to the reader domain of His-tagged protein is monitored by the singlet oxygen transfer from the Streptavidin-coated donor beads to the AlphaScreen Ni-chelate acceptor beads. Reagent:Reaction buffer:50 mM Hepes, pH7.5, 100 mM NaCl, 0.05% CHAPS, 0.1% BSA, and 1% DMSO (the final DMSO concentration may different depending on compound stock and test concentrations). Bromodomain BRD4-full length: recombinant human bromodomain containing protein 4 (bromodomain 1 and 2; aa 2–1362; Genbank Accesstion # NM_058243), expressed in *Sf9* insect cells with an N-terminal His-tag. MW = 156.5 kDa. Ligand (C-term-Biotin) Histone H4 peptide (1–21) K5/8/12/16Ac-Biotin Detection beads: PerkinElmer Donor beads: Streptavidin-coated donor beads, Acceptor beads: AlphaScreen Ni acceptor beads. Reaction procedure: (1) Deliver 2.5× BRD in wells of reaction plate except No BRD control wells. Add buffer instead. **(**2) Deliver compounds in 100% DMSO into the BRD mixture by Acoustic technology (Echo550; nanoliter range). Spin down and pre-incubation for 30 min. **(**3) Deliver 5 × Ligand. Spin and shake. **(**4) Incubate for 30 min at room temperature with gentle shaking. **(**5) Deliver 5× donor beads. Spin and shake. **(**6) Deliver 5 × acceptor beads. Spin and shake. Then gentle shaking in the dark for 60 min. **(**7) Alpha measurement (Ex/Em = 680/520–620 nm) in Enspire.

### Cell culture

Human gastric carcinoma metastatic lymph node SGC-7901 cells, human pancreatic cancer PANC-1 cells, human hepatocellular carcinoma HepG2 cells, and human lung carcinoma A549 cells were purchased from the Cell Center of the Chinese Academy of Medical Sciences (Beijing, China). Cells were cultured in Dulbecco’s modified Eagle’s medium (DMEM, Gibco, Gaithersburg, MD) or RPMI 1640 medium (Gibco, Gaithersburg, MD) supplemented with 10% foetal bovine serum (FBS) and incubated at 37 °C with 5% CO_2_ humidified atmosphere.

### MTT assay

Methyl thiazolyl tetrazolium (MTT) assay was used to detect the cell survival rate. Briefly, cells were seeded into a 96-well plate at a density of 2 × 10^4^cells/mL. Medium containing a certain concentration of compound (0, 0.47, 0.94, 1.88, 3.75, 7.50, 15.0, and 30 µM) was added into each well in a volume of 100 μL for 48 h, respectively. The cell morphology was observed by Invert/phase contrast microscopy (Nikon TE2000, Tokyo, Japan) (bar: 200 µm). Then 20 μL MTT solutions (Sigma, Shanghai, China) was added into each well and followed by incubation at 37 °C for 4 h. An aliquot of 150 μL of DMSO was further added after discarding culture medium. The crystals of formazan product were then dissolved by oscillating for 10 min. The optical density (OD) value was detected using a microplate reader (Bio-Rad, imark, Hercules, CA) at a wavelength of 570 nm. The experiments were performed in triplicate. Cell survival rate (%) = (OD of administration group − OD of blank group)/(OD of control group − OD of blank group)×100%. The value of inhibitory concentration 50 (IC_50_) was calculated by GraphPad Prism 5 software (La Jolla, CA).

### In vivo PK studies

SD female rats, 6 − 8-weeks old were selected for dosing. Three mice were randomly grouped per time point. Mice were received either a single intravenous injection of 2.5 mg/kg compound or a single oral administration of 10 mg/kg compound. Compounds were given as solutions in DMSO/PEG 200/water. Blood samples were collected from rats at 0.083, 0.25, 0.5, 1, 2, 4, 6, 8, 12, and 24 h and were further processed to obtain plasma by centrifugation at 15,000 rpm for 10 min. Plasma concentrations of the compounds were determined using the liquid chromatography − tandem mass spectrometry (LC − MS/MS) method. The pharmacokinetic parameters were calculated WinNonlin. The study was approved by the Ethical Committee of Zhejiang Academy of Medical Sciences on Use and Care of Animals, in accordance with the recommendations in the Guide for the Care and Use of Laboratory Animals.

### Computer aided drug design

The X-RAY crystal structure of BRD4 (PDB 4E96) were downloaded from Protein Data Bank (PDB) and prepared using the Protein Preparation Wizard in the Schro¨dinger suite^41^. Due to its good reproducibility of cocrystallised ligand conformations and accuracy in molecular docking and scoring^42^, Glide was selected as the molecular docking tool. A receptor grid was defined as the ligand-binding site search region based on the cocrystallised ligand and an enclosing box that was in similar in size to the cocrystallised ligand were used to capture the compounds to be docked. The cognate ligand of PDB 4E96 as well as the target compounds were prepared by LigPrep module in Schro¨dinger with the ionization stage not changed and Generate tautomers not selected. For each ligand, one low energy ring conformation was generated. The prepared compounds were submitted for molecular docking. Based on the Glide score and interactions formed between the compounds and the active site, the best conformation of each compound was conserved. Finally, the potential compounds were flexibly docked into the binding site with standard precision (SP) docking mode^43^. All the remaining parameters were kept as default settings.

### Chemistry

#### General chemistry

Commercially available reagents and anhydrous solvents were used without further purification. The crude reaction product was purified by Flash chromatography using silica gel (300–400 mesh). All reactions were monitored by TLC (thin layer chromatography), using silica gel plates with fluorescence F254 and UV light visualization. If necessary, further purification was performed on a preparative high-performance liquid chromatography (HPLC) (Waters 2545) with a C18 reverse phase column. Proton nuclear magnetic resonance (^1^H NMR) and carbon nuclear magnetic resonance (^13^C NMR) spectra were recorded on a Bruker AV-400 spectrometer at 400 MHz. Coupling constants (*J*) was expressed in hertz (Hz). Each signal is identified by its chemical shift δ expressed in parts per million (ppm). HRMS analyses were performed under ESI (electrospray ionization) using a TOF (time-of-flight) analyser in V mode with a mass resolution of 9000.

#### Synthetic procedure of compound II

To a solution of 2*H*-chromen-2-one (2.9 g, 20 mmol) in H_2_SO_4_ (10 mL) was added HNO_3_ (1.3 g, 20.1 mmol) at 0 °C then the reaction mixture was stirred at 0–5 °C for 2 h. After the reaction was completed, the reaction mixture was extracted with ethyl acetate (200 mL × 3). The organic layer was washed with brine and dried over Na_2_SO_4_.

The solid was filtered off, and the filtrate was concentrated under reduced pressure. The resulting crude product was yield 6-nitro-2*H*-chromen-2-one (**II**) without purification.

#### Synthetic procedure and characterization of compound III

To a reaction mixture of Fe powder (1.7 g, 31 mmol) and NH_4_Cl (0.8 g, 15 mmol) in EtOH (30 mL) and water (10 mL) was added 6-nitro-2*H*-chromen-2-one (1.5 g, 7.8 mmol), then the reaction mixture was stirred at 80 °C for 2 h. After the reaction was completed, the reaction mixture was cooled to room temperature (rt). The reaction mixture was extracted with ethyl acetate (100 mL × 3). The organic layer was washed with brine and dried over Na_2_SO_4_. The solid was filtered off and the filtrate was concentrated under reduced pressure. The resulting crude product was purified by silica gel chromatography with petroleum ether/ethyl acetate (5/1, v/v) to yield 6-amino-2H-chromen-2-one (**III**) (1.04 g, yield 86.7%) as a yellow solid. ^1^H NMR (400 MHz, DMSO) δ 10.34 (s, 2H), 8.19 (d, *J* = 9.6 Hz, 1H), 7.77 (s, 1H), 7.66 (dd, *J* = 8.8, 2.2 Hz, 1H), 7.52 (d, *J* = 8.9 Hz, 1H), 6.58 (d, *J* = 9.7 Hz, 1H). ^13^C NMR (101 MHz, DMSO) δ 159.98, 152.78, 143.91, 129.03, 127.01, 122.82, 119.74, 118.16, 117.81.

#### Synthetic procedure and characterization of compound 1–17

To a solution of 6-amino-2*H*-chromen-2-one (**III**) (80 mg, 0.5 mmol) and corresponding sulphonyl chloride (0.5 mmol) in DCM (5 mL) was added pyridine (0.3 mL). The mixture was stirred at rt for 2 h. The reaction mixture was extracted with DCM (30 mL × 3). The organic layer was washed with brine and dried over Na_2_SO_4_. The solid was filtered off, and the filtrate was concentrated under reduced pressure. The resulting crude product was purified by silica gel chromatography with petroleum ether/ethyl acetate (3/1, v/v) to yield **1–17.**

***N*-(2-oxo-2*H*-chromen-6-yl)benzenesulphonamide** (**1**)**:** White solids (yield 89.6%). ^1^H NMR (400 MHz, DMSO) δ 10.49 (s, 1H), 8.05 (d, *J* = 9.6 Hz, 1H), 7.80 (d, *J* = 7.6 Hz, 2H), 7.60 (dt, *J* = 26.7, 7.3 Hz, 3H), 7.47 (s, 1H), 7.31 (s, 2H), 6.48 (d, *J* = 9.6 Hz, 1H). ^13^C NMR (101 MHz, DMSO) δ 160.18, 150.79, 144.30, 139.61, 134.39, 133.51, 129.79, 127.14, 125.19, 119.92, 119.55, 117.70, 117.33. HRMS (ESI) calcd. for C_11_H_8_N_7_O_2_S ([M + H]^+^) 302.0460, found 302.0480.

***N*-(2-oxo-2*H*-chromen-6-yl)cyclohexanesulphonamide** (**2**)**:** Yellow solids (yield 92.3%). ^1^H NMR (400 MHz, DMSO) δ 9.97 (s, 1H), 8.09 (d, *J* = 9.6 Hz, 1H), 7.56 (d, *J* = 2.4 Hz, 1H), 7.45 (dd, *J* = 8.9, 2.5 Hz, 1H), 7.39 (d, *J* = 8.9 Hz, 1H), 6.50 (d, *J* = 9.6 Hz, 1H), 3.03 (t, *J* = 10.2 Hz, 1H), 2.05 (d, *J* = 11.5 Hz, 2H), 1.76 (d, *J* = 12.9 Hz, 2H), 1.59 (d, *J* = 12.0 Hz, 1H), 1.43 (m, 2H), 1.18 (m, 3H). ^13^C NMR (101 MHz, DMSO) δ 160.31, 150.34, 144.55, 135.38, 124.39, 119.63, 118.65, 117.74, 117.23, 59.50, 26.45, 25.19, 24.77. HRMS (ESI) calcd. for C_15_H_18_NO_4_S ([M + H]^+^) 308.0957, found 308.0952.

***N*-(2-oxo-2*H*-chromen-6-yl)cyclopentanesulphonamide** (**3**)**:** Pink solids (yield 85.9%). ^1^H NMR (400 MHz, DMSO) δ 9.95 (s, 1H), 8.10 (d, *J* = 9.6 Hz, 1H), 7.57 (d, *J* = 2.3 Hz, 1H), 7.48–7.37 (m, 2H), 6.50 (d, *J* = 9.7 Hz, 1H), 3.64–3.49 (m, 1H), 1.94–1.85 (m, 4H), 1.65 (m, 4H), 1.55 (m, 4H). ^13^C NMR (101 MHz, DMSO) δ 160.31, 150.53, 144.52, 135.20, 124.93, 119.63, 119.25, 117.73, 117.23, 60.21, 27.77, 25.87. HRMS (ESI) calcd. for C_14_H_16_NO_4_S ([M + H]^+^) 294.0800, found 294.0794.

***N*-(2-oxo-2*H*-chromen-6-yl)propane-1-sulphonamide** (**4**)**:** Pink solids (yield 88.4%). ^1^H NMR (400 MHz, DMSO) δ 9.98 (s, 1H), 8.10 (d, *J* = 9.5 Hz, 1H), 7.55 (s, 1H), 7.42 (q, *J* = 8.9 Hz, 2H), 6.51 (d, *J* = 9.5 Hz, 1H), 3.23–3.00 (m, 2H), 1.72 (m, 2H), 0.96 (t, *J* = 7.3 Hz, 3H) ^13^C NMR (101 MHz, DMSO) δ 160.31, 150.51, 144.51, 135.14, 124.68, 119.65, 118.96, 117.78, 117.25, 52.86, 17.30, 12.99. HRMS (ESI) calcd. for C_12_H_14_NO_4_S ([M + H]^+^) 268.0644, found 268.0635.

***N*-(2-oxo-2*H*-chromen-6-yl)butane-1-sulphonamide** (**5**)**:** White solids (yield 93.1%). ^1^H NMR (400 MHz, DMSO) δ 9.99 (s, 1H), 8.10 (d, *J* = 9.6 Hz, 1H), 7.56 (s, 1H), 7.42 (q, *J* = 9.0 Hz, 2H), 6.51 (d, *J* = 9.6 Hz, 1H), 3.18–3.07 (m, 2H), 1.75–1.61 (m, 2H), 1.45–1.30 (m, 2H), 0.85 (t, *J* = 7.3 Hz, 3H). ^13^C NMR (101 MHz, DMSO) δ 160.29, 150.50, 144.49, 135.13, 124.66, 119.64, 118.96, 117.76, 117.24, 50.85, 25.57, 21.12, 13.88. HRMS (ESI) calcd. for C_13_H_16_NO_4_S ([M + H]^+^) 282.0800, found 282.0792.

**4-chloro-*N*-(2-oxo-2*H*-chromen-6-yl)benzenesulphonamide** (**6**)**:** Pink solids (yield 86.2%). ^1^H NMR (400 MHz, DMSO) δ 10.55 (s, 1H), 8.06 (d, *J* = 9.6 Hz, 1H), 7.76 (d, *J* = 8.5 Hz, 2H), 7.64 (d, *J* = 8.5 Hz, 2H), 7.46 (s, 1H), 7.31 (m 2H), 6.49 (d, *J* = 9.6 Hz, 1H). ^13^C NMR (101 MHz, DMSO) δ 160.16, 151.00, 144.31, 138.42, 134.03, 129.98, 129.10, 125.53, 120.36, 119.63, 117.81, 117.37. HRMS (ESI) calcd. for C_15_H_11_NO_4_SCl ([M + H]^+^) 336.0097, found 336.0085.

**4-methoxy-*N*-(2-oxo-2*H*-chromen-6-yl)benzene sulphonamide (7):** Light pink solids (yield 72.8%). ^1^H NMR (400 MHz, DMSO) δ 10.34 (s, 1H), 8.05 (d, *J* = 9.6 Hz, 1H), 7.71 (d, *J* = 8.7 Hz, 2H), 7.46 (s, 1H), 7.35–7.21 (m, 2H), 7.06 (d, *J* = 8.7 Hz, 2H), 6.47 (d, *J* = 9.6 Hz, 1H), 3.80 (s, 3H). ^13 ^C NMR (101 MHz, DMSO) δ 162.98, 160.21, 150.66, 144.37, 134.69, 131.20, 129.39, 125.01, 119.57, 117.66, 117.28, 114.90, 56.00. HRMS (ESI) calcd. for C_16_H_14_NO_5_S ([M + H]^+^) 332.0593, found 332.0586.

**4-(*tert*-butyl)-*N*-(2-oxo-2*H*-chromen-6-yl)benzene sulphonamide (8):** Pink solids (yield 85.3%). ^1^H NMR (400 MHz, DMSO) δ 10.48 (s, 1H), 8.04 (d, *J* = 9.6 Hz, 1H), 7.72 (d, *J* = 8.3 Hz, 2H), 7.57 (d, *J* = 8.4 Hz, 2H), 7.48 (s, 1H), 7.31 (s, 2H), 6.47 (d, *J* = 9.6 Hz, 1H), 1.25 (s, 9H). ^13 ^C NMR (101 MHz, DMSO) δ 160.19, 156.48, 150.60, 144.36, 137.03, 134.62, 127.03, 126.65, 124.70, 119.50, 119.29, 117.73, 117.33, 35.32, 31.16. HRMS (ESI) calcd. for C_19_H_20_NO_4_S ([M + H]^+^) 358.1113, found 358.1109.

**3-cyano-*N*-(2-oxo-2*H*-chromen-6-yl)benzene sulphonamide (9):** Light pink solids (Yield 87.9%). ^1^H NMR (400 MHz, DMSO) δ 10.67 (s, 1H), 8.23 (s, 1H), 8.18 – 8.00 (m, 3H), 7.80 (t, *J* = 7.9 Hz, 1H), 7.49 (d, *J* = 2.2 Hz, 1H), 7.31 (dt, *J* = 8.9, 5.6 Hz, 2H), 6.50 (d, *J* = 9.6 Hz, 1H). ^13 ^C NMR (101 MHz, DMSO) δ 160.14, 151.14, 144.27, 140.80, 137.18, 133.61, 131.57, 131.35, 130.72, 125.68, 120.59, 119.68, 117.87, 117.39, 113.08. HRMS (ESI) calcd. for C_16_H_11_N_2_O_4_S ([M + H]^+^) 327.0440, found 327.0429.

**2-chloro-*N*-(2-oxo-2*H*-chromen-6-yl)benzenesulphonamide (10):** Light pink solids (Yield 91.0%). ^1^H NMR (400 MHz, DMSO) δ 10.86 (s, 1H), 8.07 (m, 2H), 7.70 – 7.60 (m, 2H), 7.53 (t, *J* = 6.1 Hz, 1H), 7.48 (s, 1H), 7.35 (m, 2H), 6.47 (d, *J* = 9.6 Hz, 1H). ^13^C NMR (101 MHz, DMSO) δ 160.13, 150.63, 144.19, 136.68, 135.21, 133.78, 132.36, 132.08, 131.21, 128.21, 124.37, 119.55, 118.98, 117.76, 117.40. HRMS (ESI) calcd. for C_11_H_7_N_7_O_2_SCl ([M + H]^+^) 336.0070, found 336.0091.

**2-methoxy-*N*-(2-oxo-2*H*-chromen-6-yl)benzenesulphonamide (11):** Light pink solids (Yield 84.9%). ^1^H NMR (400 MHz, DMSO) δ 10.20 (s, 1H), 8.01 (d, *J* = 9.6 Hz, 1H), 7.78 (d, *J* = 7.8 Hz, 1H), 7.56 (t, *J* = 7.9 Hz, 1H), 7.42 (s, 1H), 7.31 (m, 2H), 7.18 (d, *J* = 8.4 Hz, 1H), 7.03 (t, *J* = 7.6 Hz, 1H), 6.44 (d, *J* = 9.6 Hz, 1H), 3.90 (s, 3H). ^13 ^C NMR (101 MHz, DMSO) δ 160.22, 156.81, 150.43, 144.33, 135.68, 134.70, 130.73, 126.50, 124.63, 120.59, 119.37, 119.10, 117.48, 117.24, 113.32. HRMS (ESI) calcd. for C16 H14 N O5 S ([M + H]^+^) 332.0593, found 332.0587.

**2,4-dichloro-*N*-(2-oxo-2*H*-chromen-6-yl)benzenesulphonamide (12):** Yellow solids (Yield 83.1%). ^1^H NMR (400 MHz, DMSO) δ 10.92 (s, 1H), 8.05 (d, *J* = 8.8 Hz, 2H), 7.87 (s, 1H), 7.61 (d, *J* = 10.4 Hz, 1H), 7.45 (s, 1H), 7.33 (s, 2H), 6.48 (d, *J* = 9.6 Hz, 1H). ^13 ^C NMR (101 MHz, DMSO) δ 180.07, 150.82, 144.22, 139.20, 135.73, 133.45, 133.38, 132.45, 131.90, 128.47, 124.68, 119.82, 119.38, 117.86, 117.45. HRMS (ESI) calcd. for C_15_H_10_NO_4_SCl_2_ ([M + H]^+^) 369.9708, found 369.9694.

**4-chloro-2-methoxy-N-(2-oxo-2H-chromen-6-yl)benzenesulphonamide (13):** Pink solids (yield 81.9%). ^1^H NMR (400 MHz, DMSO) δ 10.37 (s, 1H), 8.05 (d, *J* = 9.6 Hz, 1H), 7.71 (d, *J* = 2.6 Hz, 1H), 7.64 (dd, *J* = 8.9, 2.5 Hz, 1H), 7.44 (s, 1H), 7.31 (s, 2H), 7.24 (d, *J* = 8.9 Hz, 1H), 6.46 (d, *J* = 9.6 Hz, 1H), 3.91 (s, 3H). ^13^C NMR (101 MHz, DMSO) δ 160.19, 155.71, 150.74, 144.32, 135.23, 134.16, 129.72, 128.03, 124.97, 124.15, 119.55, 117.58, 117.33, 115.49, 57.09. HRMS (ESI) calcd. for C_16_H_13_NO_5_SCl ([M + H]^+^) 366.0203, found 366.0193.

***N*-(2-oxo-2*H*-chromen-6-yl)thiophene-2-sulphonamide (14):** Light pink solids (yield 90.5%). ^1^H NMR (400 MHz, DMSO) δ 10.61 (s, 1H), 8.08 (d, *J* = 9.6 Hz, 1H), 7.91 (d, *J* = 4.9 Hz, 1H), 7.58 (d, *J* = 3.5 Hz, 1H), 7.52 (s, 1H), 7.39–7.28 (m, 2H), 7.1–7.09 (m, 1H), 6.49 (d, *J* = 9.6 Hz, 1H). ^13^C NMR (101 MHz, DMSO) δ 160.19, 151.04, 144.33, 139.93, 134.11, 133.13, 128.18, 125.42, 120.28, 119.57, 117.64, 117.36. HRMS (ESI) calcd. for C_13_H_10_NO_4_S_2_ ([M + H]^+^) 308.0051, found 308.0044.

***N*-(2-oxo-2*H*-chromen-6-yl)naphthalene-2-sulphonamide (15):** Light pink solids (yield 83.5%). ^1^H NMR (400 MHz, DMSO) δ 10.65 (s, 1H), 8.52 (s, 1H), 8.14 (t, *J* = 8.9 Hz, 2H), 8.09–7.97 (m, 2H), 7.85 (d, *J* = 8.7 Hz, 1H), 7.68 (m, 2H), 7.55 (s, 1H), 7.34 (m 2H), 6.47 (d, *J* = 9.6 Hz, 1H). ^13^C NMR (101 MHz, DMSO) δ 160.15, 150.77, 144.24, 136.63, 134.75, 134.37, 132.00, 130.00, 129.70, 129.48, 128.54, 128.22, 125.16, 122.44, 119.90, 119.55, 117.70, 117.30. HRMS (ESI) calcd. for C_19_H_14_NO_4_S ([M + H]^+^) 352.0644, found 352.0634.

***N*-(2-oxo-2*H*-chromen-6-yl)-2,3-dihydrobenzofuran-5-sulphonamide (16):** Light pink solids (yield 91.9%). ^1^H NMR (400 MHz, DMSO) δ 10.28 (s, 1H), 8.05 (d, *J* = 9.6 Hz, 1H), 7.63 (s, 1H), 7.53 (d, *J* = 9.5 Hz, 1H), 7.44 (s, 1H), 7.34–7.24 (m, 2H), 6.86 (d, *J* = 8.5 Hz, 1H), 6.46 (d, *J* = 9.6 Hz, 1H), 4.60 (t, *J* = 8.8 Hz, 2H), 3.20 (t, *J* = 8.8 Hz, 2H). ^13^C NMR (101 MHz, DMSO) δ 163.7, 160.24, 150.54, 144.40, 134.77, 131.21, 129.41, 128.67, 124.87, 124.54, 119.46, 117.65, 117.27, 109.55, 72.69, 28.83. HRMS (ESI) calcd. for C_17_H_14_NO_5_S ([M + H]^+^) 344.0593, found 344.0586.

***N*-(4-methoxyphenyl)-2-oxo-2*H*-chromene-6-sulphonamide (17):** Light purple solids (yield 77.2%). ^1^H NMR (400 MHz, DMSO) δ 10.03 (s, 1H), 8.16 (d, *J* = 9.6 Hz, 1H), 8.11 (s, 1H), 7.85 (d, *J* = 7.0 Hz, 1H), 7.56 (d, *J* = 8.7 Hz, 1H), 7.00 (d, *J* = 8.8 Hz, 2H), 6.81 (d, *J* = 8.8 Hz, 2H), 6.60 (d, *J* = 9.6 Hz, 1H), 3.67 (s, 3H). ^13^C NMR (101 MHz, DMSO) δ 159.60, 157.22, 156.17, 143.92, 135.89, 130.15, 128.08, 124.39, 119.29, 118.20, 117.96, 114.85, 55.62. HRMS (ESI) calcd. for C_16_H_13_NO_5_NaS ([M + Na]^+^) 354.0412, found 354.0398.
